# Pennsylvania Hospital—Surgical Wards—Service of Dr. Norris

**Published:** 1850-11

**Authors:** W. H. Gobrecht

**Affiliations:** Resident Surgeon


*Pennsylvania Hospital—Surgical Wards—Service of* DR. NORRIS.
*Cases discharged from Dec. 1st,* 1849, *to Jan. 1st,* 1850.

| Disease. | Cured. | Average time under treatment in days | By request. | Average time in days under treatment. | Died. | Average time in days under treatment. | Tot. |
|---|---|---|---|---|---|---|---|
| Abscess - - | 3 | 11.3 | 1 | 7 | 0 | 0 | 4 |
| Burns - - | 1 | 4 | 0 | 0 | 0 | 0 | 1 |
| Cataract - - | 0 | 0 | 1 | 79 | 0 | 0 | 1 |
| Cirsocele - | 1 | 16 | 0 | 0 | 0 | 0 | 1 |
| Contusions - | 2 | 7 | 0 | 0 | 0 | 0 | 2 |
| Fistula in ano - | 1 | 63 | 0 | 0 | 0 | 0 | 1 |
| Fractures, 18 | | | | | | | |
| simple 11, viz.: | | | | | | | |
| Clavicle - | 2 | 39 | 0 | 0 | 0 | 0 | 2 |
| Condyle of humerus | 1 | 45 | 0 | 0 | 0 | 0 | 1 |
| Forearm - | 3 | 46 | 1 | 19 | 0 | 0 | 4 |
| Femur - - | 1 | 111 | 0 | 0 | 0 | 0 | 1 |
| Humerus - | 1 | 41 | 0 | 0 | 0 | 0 | 1 |
| Nose - - | 0 | 0 | 1 | 4 | 0 | 0 | 1 |
| Patella - - | 1 | 45 | 0 | 0 | 0 | 0 | 1 |
| Compound, 7, viz.: | | | | | | | |
| Fingers - - | 4 | 33.5 | 0 | 0 | 0 | 0 | 4 |
| Femur - - | 0 | 0 | 0 | 0 | 1 | 6 | 1 |
| Cranium - | 0 | 0 | 0 | 0 | 1 | 5 | 1 |
| Leg - - | 2* | 257.5 | 0 | 0 | 2 | 6.5 | 4 |
| Toes - - | 1 | 46 | 0 | 0 | 0 | 0 | 1 |
| Gonorrhœa - | 1 | 35 | 0 | 0 | 0 | 0 | 1 |
| Hydrocele - | 2 | 20 | 0 | 0 | 0 | 0 | 2 |
| Inflammat'n of hand | 1 | 25 | 1 | 1 | 0 | 0 | 2 |
| " knee | 1 | 54 | 1 | 150 | 0 | 0 | 2 |
| " foot | 1 | 24 | 0 | 0 | 0 | 0 | 1 |
| Necrosis - - | 1 | 17 | 0 | 0 | 0 | 0 | 1 |
| Ophthalmia - | 1 | 11 | 0 | 0 | 0 | 0 | 1 |
| Paronychia - | 1 | 49 | 0 | 0 | 0 | 0 | 1 |
| Porrigo scutulata | 1 | 28 | 0 | 0 | 0 | 0 | 1 |
| Sub-luxation - | 1 | 11 | 0 | 0 | 0 | 0 | 1 |
| Syphilis - - | 6 | 39.3 | 2 | 8 | 1 | 4 | 9 |
| Ulcer - - | 2 | 29.5 | 1 | 25 | 0 | 0 | 3 |
| Wounds 7 viz.: | | | | | | | |
| Gunshot - | 1 | 3 | 2 | 41.5 | 0 | 0 | 3 |
| Incised - | 1 | 104 | 0 | 0 | 0 | 0 | 1 |
| Lacerated - | 2† | 27 | 0 | 0 | 0 | 0 | 2 |
| | 47 | | 11 | | 5 | | 63 |

* One of these cured by amputation, after a long continued effort to save it; but in the end the deformity was so great, that the limb was useless. The injury was originally caused by the patient leaping from the box of a carriage whilst the vehicle was in rapid motion, thus producing a compound fracture of the leg just above the ankle joint, both bones protruding and striking the ground.

† One was a lacerated wound of the cornea.

W. H. GOBRECHT, Resident Surgeon.

